# Spotting fake news: a qualitative review of misinformation and conspiracy theories in acne vulgaris

**DOI:** 10.1111/ced.15222

**Published:** 2022-05-28

**Authors:** Cathal O'Connor, Ciara O'Grady, Michelle Murphy

**Affiliations:** ^1^ Department of Dermatology South Infirmary Victoria University Hospital Cork Ireland; ^2^ Department of Medicine University College Cork Cork Ireland

## Abstract

Acne vulgaris is an extremely common disorder of the pilosebaceous unit, typically manifest as a highly visible facial and upper trunk dermatosis, with teenagers most frequently affected. This cohort is markedly susceptible to misinformation, given their impressionable age, distress about their appearance and high internet usage. This study aimed to assess the content of acne‐related misinformation available online. A formal review of PubMed was performed in March 2022, using the terms ‘acne’ AND ‘misinformation’ OR ‘disinformation’ OR ‘conspiracy theory’, along with an informal Google search using combinations of these terms, and further targeted searches on TikTok, Twitter, Facebook and Instagram. Key themes of acne‐related misinformation included diet and other ‘causes’ of acne, unconventional acne ‘cures’ and a distrust of conventional acne treatments. Websites promoting misinformation were frequently affiliated with companies selling products that promised to cure acne, often in a remarkably short time. Dermatologists should be aware of the nature of acne‐related misinformation available online and be prepared to counter it with scientific principles and facts.

## Graphical abstract

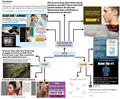

Acne vulgaris is a multifactorial disorder of the pilosebaceous unit with predominantly facial involvement, peak incidence during adolescence and a significant psychosocial impact on patients.^1^ Misinformation in healthcare is a global challenge, and young people often seek advice about acne online and on social media, leaving them particularly vulnerable to misinformation.^2^ The aim of this study was to assess misinformation around acne available online.

## Report

A literature research search was conducted on PubMed using the terms ‘acne’ AND ‘misinformation’ OR ‘disinformation’ OR ‘conspiracy theory’. This search identified 1024 abstracts, which were reviewed for suitability by 2 of the authors (COG and COC), who deemed 5 papers appropriate for inclusion, as they contained content specific to acne‐related misinformation (Table 1). An informal Google search was also conducted, using combinations of the terms ‘acne’ and ‘misinformation’, ‘disinformation’ and ‘conspiracy theories’, and further targeted searches were performed on TikTok, Twitter, Facebook and Instagram. These searches were carried out in March 2022.

**Table 1:** Studies identified following PubMed search as containing data on content of misinformation related to acne.

Findings	Reference
YouTube videos on acne are inaccurate and low quality	Borba AJ, Young PM, Read C, Armstrong AW. Engaging but inaccurate: a cross‐sectional analysis of acne videos on social media from non‐health care sources. *J Am Acad Dermatol* 2020; **83:** 610–12
Misinformation regarding dietary, face‐washing and UV‐exposure behavioural modifications in acne management are common	Magin P, Pond D, Smith W, Watson A. A systematic review of the evidence for ‘myths and misconceptions’ in acne management: diet, face‐washing and sunlight. *Fam Pract* 2005; **22:** 62–70
Patients with acne had very poor knowledge of the aetiology of acne	Yorulmaz A, Yalcin B. Myths, perceptions and practices in acne: a study on adolescents and young adults. *Curr Health Sci J* 2020; **46:** 111–16
Some patients had good conceptions of certain aspects of acne, such as the influence of hormones or food, whereas others had misunderstandings about the effects of poor hygiene on acne. Friends and websites were the most common information resources used by patients	Wisuthsarewong W, Nitiyarom R, Kanchanapenkul D *et al*. Acne beliefs, treatment‐seeking behaviours, information media usage and impact on daily living activities of Thai acne patients. *J Cosmet Dermatol* 2020; **19:** 1191–5
Social media‐influenced acne treatment advice is prevalent, especially among women, adolescents and young adults. This treatment advice frequently does not align with AAD guidelines, with notably 40% of respondents choosing dietary modification for acne management. These results suggest that dermatologists should inquire about use of acne‐treatment advice from social media and directly address misinformation	Yousaf A, Hagen R, Delaney E *et al*. The influence of social media on acne treatment: a cross‐sectional survey. *Pediatr Dermatol*. 2020; **37:** 301–4

AAD, American Academy of Dermatology; UV, ultraviolet.

**Figure 1:**
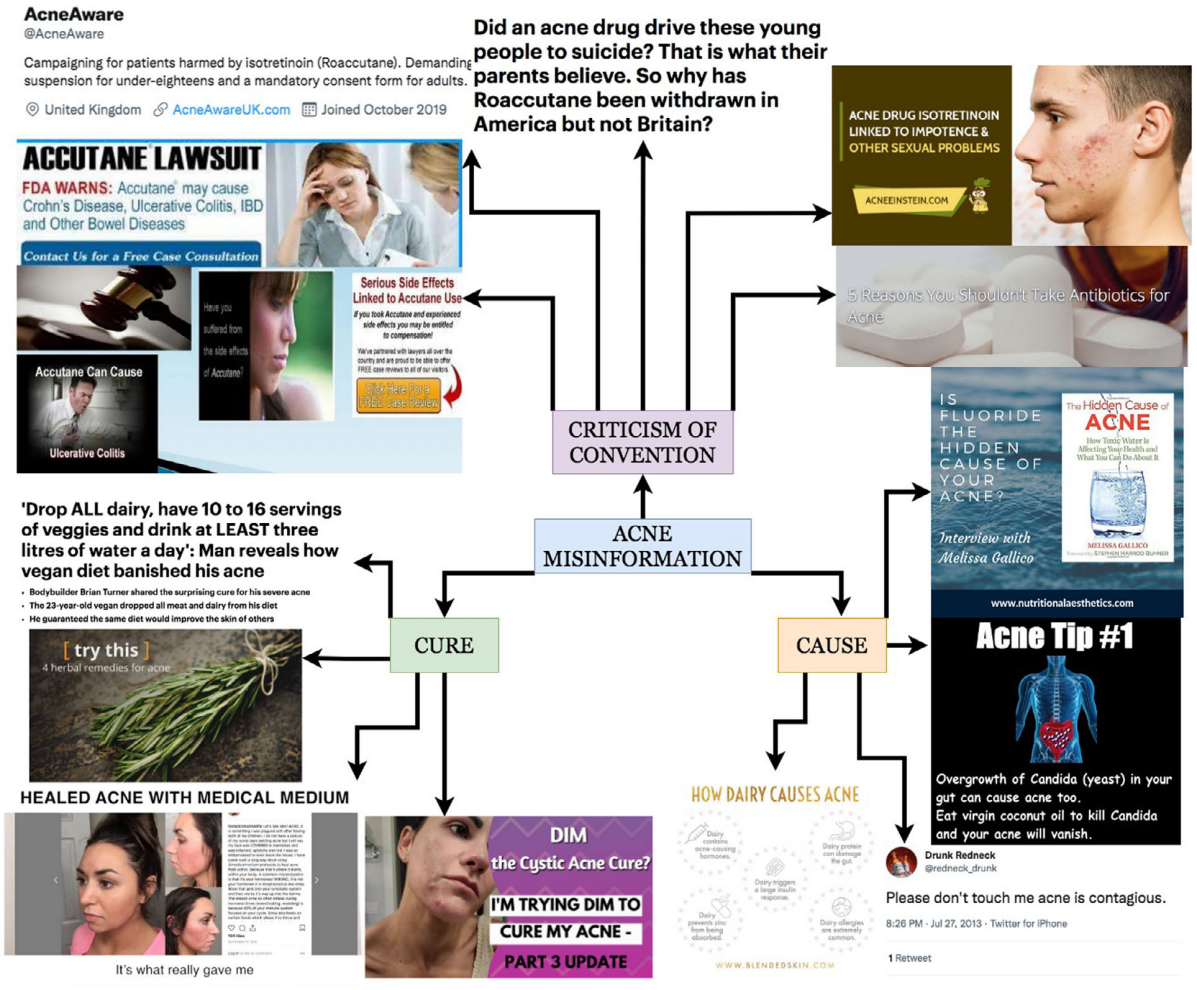
Flowmap showing content of online misinformation related to acne. Sources from top right: acneeinstein.com, acneeinstein.com, skinwellnesspro.com, pinterest.com, twitter.com, blendedskin.com, youtube.com, medicalmedium.com, healthline.com, dailymail.co.uk, pt.slideshare.net, twitter.com, dailymail.co.uk. [Colour figure can be viewed at wileyonlinelibrary.com]

Key themes of acne‐related misinformation included diet and other ‘causes’ of acne, unconventional acne ‘cures’ and a distrust of conventional acne treatments (Fig. 1). Alleged causes of acne included multiple foods, poor hygiene, systemic infections and fluoridated water. Several ‘miracle cures’ for acne were identified, including veganism, nutritional supplements and branded creams, often claiming to rapidly eradicate severe acne refractory to standard therapy. Conventional acne treatments, most often in the form of antibiotics and oral isotretinoin, frequently had negative connotations. In particular, the controversial relationship between isotretinoin and depression featured strongly, with the dug being referred to as an ‘extreme’, ‘last resort’ and ‘radical’ treatment.

In terms of ‘causes’, diet was frequently mentioned, with dairy products most often cited. Although previous studies have shown there may be a very modestly increased risk of acne with higher milk intake,^3^ there is no evidence that it causes acne and there are potential consequences to dairy avoidance, such as calcium and vitamin D deficiency. Other alleged dietary causes included chocolate and ‘greasy’ or high‐fat foods. The strongest evidential association between diet and acne is with a high glycaemic load diet (i.e. high‐sugar foods), thought to increase levels of insulin‐like growth factor‐1, which activates keratinocytes and sebocytes, leading to increased acne flares.^4^ However, interestingly, low‐glycaemic diets were rarely promoted on online forums. As some dietary interventions may cause harm, they should be implemented carefully, and a low‐glycaemic diet is a reasonable modification that may have other health benefits such as weight loss. Acne was occasionally attributed to poor hygiene (which may compound patient distress), usually by companies selling facial washes or antiseptic products. Fortunately, acne was rarely reported to be contagious. Systemic infections were also claimed to cause acne, particularly *Streptococcus* and gut yeast overgrowth, with ‘*Candida* cleanses’ championed by wellness practitioners. More extreme blogs declared that ‘the hidden cause’ of acne was fluoridated water, with no plausible scientific basis provided.

In terms of ‘cures’, veganism was frequently mentioned. Although veganism has many tangible benefits, such as reduced risk of cardiovascular disease, decreased environmental impact and ethical considerations, there is no supportive evidence for veganism in reducing acne severity.^5^ Despite this, several veganism support groups claim that veganism can rapidly clear acne. Other ‘miracle cures’ included branded topical treatments, usually lauded in testimonials as achieving clear skin within weeks, often following supposedly failed conventional treatments. These topical treatments were often marketed as being ‘natural’ or ‘herbal’. Other ‘wonder’ treatments for pimples included toothpaste, lemon juice and garlic. Related to diet, nutritional deficiencies were frequently mentioned as a cause of acne, with expensive supplements recommended to achieve clear skin. Ironically, some supplements can actually precipitate or aggravate acne, for example vitamin B6/B12, iodine and of course supplements adulterated with anabolic steroids. Examples of recommended supplements included zinc, magnesium and probiotics. Diindolylmethane (DIM), a compound found in cruciferous vegetables, was touted as a ‘hormone balancer’, which blocks androgen receptors and promotes ‘production of good estrogen’. Although DIM has been studied as a chemoprotective and anti‐inflammatory agent *in vitro*, some reports that ascribed anticancer effects to it have been retracted due to data falsification,^6^ and there are no clinical studies assessing use of DIM in acne. A ‘medical medium’ also claimed to cure your acne if you bought his book for $16.99.

Conventional treatments were heavily criticized in many posts. Antibiotics were referred to as ‘pollutants’, with ‘health‐ruining’ effects on skin and gut microbiomes. Isotretinoin was vilified as toxic with profound and irreversible adverse effects. The controversial link with depression was frequently cited, highlighting individual reports of severe depression in patients on isotretinoin, including challenge/dechallenge responses.^7^ However, multiple meta‐analyses have found that there is no population‐level association of isotretinon with depression, and that the drug may in fact be associated with a reduction in depressive symptoms due to improved quality of life.^8^ However, although the development or worsening of depression on isotretinoin is very rare and may represent a dermatological ‘nocebo’ effect, the occurrence of idiosyncratic reactions makes it sensible to monitor patients for mood changes. The previously reported association of isotretinoin with inflammatory bowel disease (IBD) was also commonly reported online; however, earlier studies ignored prior antibiotic therapy as a potential confounder, and more recent higher‐quality reviews have confirmed that there is no increased risk of IBD with isotretinoin.^9^ Blogs also exaggerated the risk of organ injury with isotretinoin, saying that the drug often caused liver or kidney failure, whereas laboratory abnormalities are extremely rare in patients on isotretinoin.^10^ Isotretinoin is well‐known to be teratogenic when prescribed to female patients; however, this fact was extrapolated in online material to suggest that it also causes infertility, erectile dysfunction and sexual dysfunction.

The psychosocial impact that acne can have on teenagers, who may spend a considerable amount of time online, leaves them particularly vulnerable to misinformation. The highly visible nature of the disease often leaves patients yearning for a cure. This desperation, in combination with misinformation about the efficacy and safety of conventional treatments, can lead to patients embracing expensive alternative treatments peddled by charlatans. Dermatologists should be aware of the large amount of misinformation about acne that is available online, and be prepared to refute and rebut misleading health information.

**Learning points**

• Acne is an extremely common and visible dermatosis that usually affects teenagers and young people and can have a significant psychosocial burden; this group of patients is at risk of misinformed healthcare decisions due to their distress about their skin and their ready access to the internet.
• Key themes of acne‐related misinformation identified in this study included diet and other ‘causes’ of acne, unconventional acne ‘cures’ and a distrust of conventional acne treatments.
• Dietary ‘causes’ of acne included exposure to dairy products, high‐fat foods and chocolate; other alleged causes included poor hygiene, contagion, systemic infections such as *Candida* and use of fluoridated water.
• Reported ‘cures’ included veganism, ‘natural’ topical treatments and nutritional supplements such as DIM.
• Conventional treatments were heavily criticized, with antibiotics called ‘poisonous’ and isotretinoin referred to as ‘toxic’ and ‘devastating’, with exaggerated reports of depression, IBD, organ failure, infertility and sexual dysfunction.
• Dermatologists should be aware of the distress caused by acne and the type of misinformation available online and be prepared to combat fake news with evidence‐based practice.

## Conflict of interest

The authors declare that they have no conflict of interest.

## Funding

Not applicable.

## Ethics statement

Ethics approval was not applicable. The patient provided informed consent for publication of their case details and images.

## Data availability

Data are available on request from the corresponding author.
